# Supporting healthy lifestyle behaviours in families attending community playgroups: parents’ perceptions of facilitators and barriers

**DOI:** 10.1186/s12889-019-8041-1

**Published:** 2019-12-27

**Authors:** Andrea B. Fuller, Rebecca A. Byrne, Rebecca K. Golley, Stewart G. Trost

**Affiliations:** 1 Institute of Health and Biomedical Research, Queensland University of Technology, Centre for Children’s Health Research, 62 Graham St, South Brisbane, QLD 4101 Australia; 2 NHMRC Centre of Research Excellent in the Early Prevention of Obesity in Childhood, Sydney School of Public Health, Level 6, Charles Perkins Centre, University of Sydney, Sydney, Australia; 3 College of Nursing and Health Sciences, Flinders University, Adelaide, Australia

**Keywords:** Early childhood, Parenting, Feeding practices, Focus groups, Physical activity, Screen time, Sleep

## Abstract

**Background:**

Establishing healthy nutrition, activity, and sleep behaviours early in life is a key strategy in childhood obesity prevention. Parents are the primary influence on the development and establishment of obesity-related behaviours in young children. There is evidence that autonomy supporting parenting practices are crucial for the development of self-regulation and the internalisation of healthy behaviours in children. It is therefore imperative that parenting practices are targeted as part of an obesity prevention intervention. However, there is limited understanding of barriers and facilitators to parents using autonomy supporting parenting practices with their children aged 0–5 years. Therefore, the aim of the study was to identify barriers and facilitators to using autonomy supporting parenting practices. A secondary aim was to determine parent preferences in respect to an intervention program to be delivered in community playgroups.

**Methods:**

Parents were recruited through Playgroup Queensland (PGQ), a not-for-profit organisation in Brisbane, Australia, to attend a focus group during their usual playgroup session. The focus group interview guide was designed to promote discussion among the participants in respect to their shared experiences as parents of young children. The focus group transcripts were coded and analysed using qualitative content analysis. Five focus groups with parents (*n* = 30) were conducted in May 2018. Most of the participants were mothers [1], and the majority (76%) had a child at playgroup aged between 2 and 4 years.

**Results:**

The support and guidance received from other parents at playgroup was a facilitator to autonomy supporting parenting practices. Barriers included beliefs around the need to use rewards to encourage child eating, beliefs around the need for screens as babysitters, and feeling disempowered to change sleep behaviours. Parents were enthusiastic about a potential program that would leverage off the existing playgroup support networks, but they did not want to be “educated”, or to lose their “playgroup time” to an intervention. Rather they wanted strategies and support to deal with the frustrations of food, screen and sleep parenting.

**Conclusion:**

These results will be used to inform the development of a childhood obesity prevention intervention to be delivered in a community playgroup setting.

## Background

The global obesity epidemic is recognised as a critical public health issue that needs to be tackled in early childhood [2]. Unhealthy eating behaviours, physical inactivity, and inadequate sleep increase obesity risk, and these behaviours often cluster together in children and adolescents to further increase that risk [3]. Obesity-related behaviours develop during the early years of life, so interventions need to target these behaviours before they become established [4]. Parents and other primary caregivers are the major influencers in the development of the behaviours through their parenting practices [5].

Parenting practices are the way parents behave or what they do in the performance of their parental duties [6]. Parenting practices include the setting of rules, explaining rules, restriction of certain foods, providing structure, setting limits on screen-time or taking children to sporting activities [7]. There is increasing evidence that certain parenting practices in each of the obesity-related behavioural domains may increase or decrease the risk of childhood obesity [8, 9].

Autonomy supporting parenting practices are crucial for the development of self-regulation and the internalisation of healthy behaviours in children [10]. Examples of autonomy supporting parenting practices include using feeding practices that support the child to recognise their own hunger and satiety cues [11], providing support for physical activity [12], providing rules and limits around screen time [13], and establishing bedtime routines [14]. In order to effectively use autonomy supporting parenting practices, parents require skills and knowledge, and the confidence to use them [9, 15]. However, there is limited understanding of the barriers and facilitators to parents using autonomy supporting parenting practices with their children aged 0–5 years [16].

General parenting programs provide advice and strategies for dealing with challenging child behaviours. In school-age children, there are some examples where general parenting programs have been applied to address obesity-related behaviours directly [17–19]. However, the majority of interventions target only one or two behavioural domains, usually nutrition and/or physical activity [20, 21], and few have targeted sleep behaviours or sleep parenting practices [20]. Interventions that have included sleep behaviours generally target parents of infants, particularly first-time mothers [22]. Few interventions have targeted all four obesity-related behaviours [23].

Childhood obesity prevention interventions targeting parents of children under the age of 2 are typically home-based, or delivered in a primary care setting [24]. The majority that focus on toddlers and preschoolers have been implemented in Early Childcare and Care settings [25], with some parental involvement as an adjunct to the main program [26]. Few interventions that target parents of young children have been delivered in a community group setting [23]. A unique advantage of an intervention delivered to existing parent groups is that they are already a source of support for parents of young children [27]. However, little is known about the potential for targeting parenting practices, knowledge, skills and confidence in this setting [4].

In Australia, a widely available parent group is the community playgroup, a place where parents and their young children meet informally, once or twice a week for 2–3 h at a community venue, for social interaction and for the children to play [28]. Community playgroups are run by volunteer parents and are open to all parents and carers of children aged from birth to 5 years [1]. The vision and values of community playgroup are to nurture young children and support the wellbeing of families [1]. The organisation recognises parents as first teachers, and provides an environment that encourages peer support and family bonding [29]. The philosophy behind the playgroup values, therefore, creates a synergy with childhood obesity prevention initiatives that focus on supporting positive and effective parenting practices. In addition, the reach of playgroups is vast. Across Australia, there are over 8000 Community playgroups operating in 80% of postcodes, and they are made up of families with a diverse range of cultural, social and economic backgrounds [1]. Despite community playgroups providing a unique opportunity to reach parents with young children, few obesity prevention interventions have been delivered in this setting [30].

The aim of this study, therefore, was to inform the design of a childhood obesity prevention intervention in the community playgroup setting by 1) identifying the barriers and facilitators in respect to using parenting practices that support the development of healthy obesity-related behaviours in their child; and 2) determine what parents would find acceptable in terms of delivery mode and timing of an intervention.

## Methods

### Study design

Focus groups with parents attending community playgroups were conducted at their usual playgroup location and time. A focus group methodology was selected because it was expected that the group discussion would provide richer data than individual interviews, as shared experiences and understandings encourage participants to openly discuss their challenges as parents [31].

### Theoretical framework

**Fig. 1:**
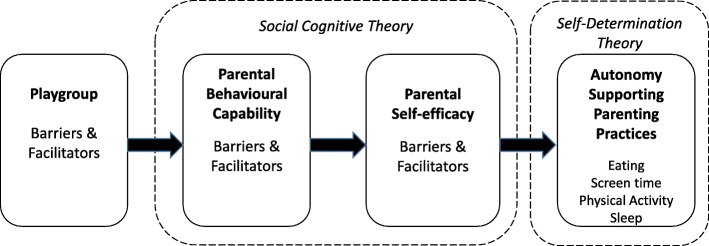
Conceptual framework for the focus groups

A deductive content analysis approach [32], was used to develop the semi-structured topic guide, using a conceptual framework (Fig. 1) based on Social Cognitive Theory and Self-determination Theory [33–35]. The framework, therefore, encompassed facilitators and barriers with respect to parent’s knowledge and skills around autonomy supporting parenting practices (behavioural capability) and their confidence to use them regularly (self-efficacy).

### Recruitment and consent

Community playgroups operating in the greater Brisbane metropolitan area were invited to participate in the research project via a newsletter from PGQ. The newsletter stated that focus groups were being conducted to gather information on parenting in respect to child eating, active play, screen time and sleep. The focus groups would also seek parents views on how a program that supports parents around those child behaviours could be delivered at playgroup. Seven playgroups expressed an interest in taking part in the focus groups, and five of these were recruited for the focus groups. The other two playgroups expressed their interest after the focus groups for the other five had been conducted, and were not required as it was deemed that no further insights would be gained from conducting further focus groups. All parents attending playgroup on the day of the focus group were invited to participate and provide informed consent. Participants were provided with an information sheet about the study and also given a verbal explanation of why the focus groups were being conducted before each discussion commenced.

### Data collection

Focus groups were conducted in May 2018 by two researchers (AF and RB). The discussions were guided by the topic guide, which included questions around what parents enjoyed about coming to playgroup, where they accessed information about healthy child behaviours, barriers to encouraging healthy behaviours, and strategies they used to influence healthy behaviours in their child/ren. Parents were asked about behaviours around eating, screen-time, active play and sleep. They were also asked whether they would be interested in a healthy lifestyle program and to consider how it might work at their playgroup. This included discussing options for the number, timing and length of intervention sessions; and the preferred characteristics of a potential facilitator. The topic guide is provided as supplementary material [see Additional file 1]. Participants also completed a survey that measured demographic characteristics (age, relationship to their children at playgroup, children’s age, work status, education, and whether they were born in Australia).

All focus groups were conducted on site, during playgroup time, and were audio recorded and transcribed verbatim by AF. RB took notes in respect to general impressions, and noted when participants left (and returned) to the group. Children participated in their usual playgroup activities within sight of their parents taking part in the focus group or under the supervision of other adults. AF and RB debriefed after each focus group, and additional reflections were documented. The debrief after the first focus group also considered whether the data obtained addressed the research questions and how the next group moderation could be improved. No changes to the topic guide were made, however the order in which the topics were raised varied slightly in each focus group according to how the discussion progressed.

### Data analysis

Qualitative content analysis was used to analyse the focus group data, using NVivo 12 (QSR International Pty. Ltd.). The aim was not to search for underlying meanings via latent content, or produce results that are highly interpretive. Rather it was to take the words of the participants at “face value” [36] in order to identify recurring themes that encapsulated the parenting priorities of the participants, and to develop an intervention that met the expressed needs of parents at playgroup.

The main categories of the coding matrix and initial codes were deductively determined from the conceptual framework (Fig. 1) and research questions. Although a deductive approach was used to develop these main categories and the a priori codes, the overall analysis of the focus group data was both deductive and inductive. Immersion in the data by the primary researcher (as moderator and transcriber) shaped some of the a priori codes. The main categories were the SCT constructs of parental behavioural capability and parental self-efficacy and the SDT construct of autonomy promoting parenting practices. The generic categories were the facilitators and barriers within each main category. When participants discussed parenting practices, knowledge of guidelines or knowledge of supportive parenting practices was coded as a facilitator of behavioural capability. If a comment demonstrated a lack of knowledge, then it was coded to a barrier to behavioural capability. Comments that inferred confidence in parenting, optimism, or receiving support as a parent were coded as facilitators of parental self-efficacy. Comments related to feeling stressed, tired or guilty were coded a barriers. The coding of facilitators and barriers to autonomy promoting parenting practices centred around comments about positive or negative factors in the home environment, family influences, support from peers, parent intentions to use supportive parenting practices, and specific examples of either autonomy-promoting or non-autonomy-promoting parenting practices.

AF and RB independently coded one of the transcripts and the coding frame was updated to reflect shared understandings of codes. During coding of all of the transcripts by AF, a more inductive approach was used to develop sub-categories and to further refine the coding frame based on the data. These sub-categories and the associated codes were the specific facilitators and barriers discussed by the participants. Playgroup environment codes were developed inductively from the transcripts, and grouped as facilitators or barriers to an intervention delivered in this setting.

## Results

### Participant characteristics

Five playgroups agreed to take part in a focus group, which ranged in length between 40 and 60 min. The number of participants in each focus group varied from 4 to 7. Twenty-eight of the 30 participants were mothers. The median age of the children was 24.0 months (IQR = 12.0 months). Other characteristics of the participants are shown in Table 1.

**Table 1:** Demographic characteristics of the focus group participants

Variable		Participants (*n* = 30)	
		N	%
Relationship to child/ren	Mother	28	93.3
	Father	1	3.3
	Grandmother	1	3.3
Age of parent/carer	Under 30 years	4	13
	30–35 years	11	37
	36 years or older	15	50
Education	University education	15	50
	TAFE or trade	12	40
	Secondary school	3	10
Employment status	Not in paid employment	15	50
	Part-time employment	12	40
	Full-time employment	3	10
Born in Australia	Yes	23	77
Number of children per parent/carer at playgroup	One	20	67
	Two	9	30
	Three	1	3
Age of child at playgroup (*n* = 41)	Under 24 months	10	24
	24–35 months	17	42
	36–47 months	7	17
	48–60 months	7	17

### Facilitators and barriers to autonomy supporting parenting practices

Participants talked openly about their positive experiences as well as the many challenges around parenting of young children. Topics that were consistently raised in the discussions included issues around food refusal, electronic media, and child sleep. In general, parents were less concerned about their child’s level of physical activity as most perceived their child to be sufficiently active. Two main themes emerged in relation to facilitators of autonomy supporting parenting practices: 1) Parents are confident in their knowledge but want strategies; and 2) Support from peers at playgroup is highly valued. Two main themes emerged in relation to barriers to autonomy supporting parenting practices: 1) Lack of empowerment to influence child preferences; and 2) Stress, tiredness or lack of time can make parenting a challenge.

*Facilitator Theme 1: Parents are confident in their knowledge but want strategies.*

Participants were generally confident that they had the knowledge around healthy behaviours for young children. Parents reported that they were confident that they knew what their child should (and should not) be eating. Although specific guidelines were not discussed, they were also aware that screen time should be limited, that physical activity is important for health, and that children need a certain number of hours of sleep each night. However, despite this awareness, parents indicated that they struggled to apply that knowledge. They wanted guidance on how to translate their knowledge into effective strategies. Parents specifically requested help with their child’s “fussy eating”.

*“I would ideally like to encourage a healthy diet … encouraging is one thing, having it actually happen is another thing.” Parent, FG2.*

Across all focus groups, parents expressed strong beliefs about what constituted a healthy diet. The importance of vegetables, in particular, was a common discussion point. The main focus was on the evening meal, and the importance of eating everything on the plate. Some parents reported offering rewards or bribes of highly palatable, energy dense, foods (generally chocolate or dessert) to encourage the child to finish the meal. Other common strategies were hiding vegetables within the meal, or only providing food the parent knows the child will eat. Some parents felt these tactics were good strategies to encourage adequate nutrition, whereas others were aware that the use of bribes was not ideal.

*“You’ll get a treat if you eat your food. I think that’s fine, if it gets him to eat his food.” Parent 1, FG1.*

*“It takes a lot of time, this whole eating healthy thing because you gotta hide it.” Parent 2, FG1.*

*“If you give them too much [confectionary as a bribe] you feel guilty. Because you know it’s wrong.”, FG4.*

Several parents talked about offering novel foods multiple times to their child in order to develop a liking for that food.

*“[My child] went through a fussy stage. I just kept providing the same stuff and not giving alternatives. And eventually he got over that. But for two years, he wouldn’t eat certain textures, he wouldn’t eat mixed foods. But I just kept providing the same stuff.” Parent, FG1.*

Parents discussed struggles in respect to restricting the use of screens, particularly iPads®. Some parents commented that they used diverting strategies to minimise screen time, such as suggesting the child go outside to play or engaging in an activity with their child. Other parents hid the electronic devices or put a schedule on the fridge to limit screen use to certain times. However, although most parents across the groups were aware that screen time should be limited, the majority mostly discounted this advice, either because they felt the guidelines were unnecessarily restrictive, or because they found screens a useful parenting aid. Many parents felt that screens were unavoidable in certain situations, generally in respect to using them to occupy their child in order to shower or do household chores. Some parents also commented that screens were useful to “calm” their child before bed or when they were overly active.

*“You don’t want kids around you in the kitchen, when you’re cooking. So, for them to sit down, they’re sitting there they’re calm, they’re watching TV. I don’t think it’s such a bad thing.” Parent, FG3.*

*“If they’re overtired … to make him sedate. … if I’m at that stage where he just needs to stop because he’s going crazy.” Parent, FG1.*

Some parents also felt that iPads® were necessary because children need to be familiar with them before starting school. Most agreed that, as long as the app was educational, it mitigated the potentially negative aspects of screen use.

*“They do need some screen time because the reality is that so much of the world is that these days. So if they don’t use it at all, then they fall behind other children, I think.” Parent, FG3.*

Parents did not discuss sleep recommendations or why sleep was important. They did not state they disagreed with sleep guidelines, just that they struggled to influence the amount of sleep their child received. A number of parents mentioned challenges around getting children to sleep, night waking, and early rising. The limited success of strategies they had tried was also discussed in the groups, as well as strategies that were counterproductive, such as rocking a child to sleep, or strapping them in a car seat.

*“My problem’s not getting them to bed, it’s the time he wakes up. And he wakes up during the night.” Parent, FG1.*

*“I don’t know what more information I could have done with – I read everything. It didn’t help.” Parent FG4.*

Despite the challenges expressed by the parents, there were also comments that suggested they had self-confidence in most areas of parenting. A number expressed a confidence to assess parenting information and make a decision with respect to a particular issue based on their own values and situation. Words such as “common sense” and “instinct” were used multiple times across the groups.

*“I sort of take bits and pieces from various people and books and things and just kind of make a bit of a collage of what’s best for him and for me …*” *Parent, FG5.*

*Facilitator Theme 2: Support from playgroup peers is highly valued.*

Although the frustrations and stress of parenting were a focus of the group discussions, this was tempered to a large degree by a general outlook of optimism and a belief that their parenting challenges were temporary. This attitude was facilitated by the support received from their playgroup peers, including older parents or grandparents attending the playgroup, and an attitude that “we are all in it together”. While there was some mention of mothers’ groups for infants, and support received from family and friends, the predominant source of support was from other parents at playgroup. In fact, receiving support from their peers was identified as a major reason for attending playgroup.

*“I think also sharing stories, talking to other mums and sharing what’s happened during the week, and then going hey, you’re not the only one.” Parent, FG1.*

*“What we all bring is different experiences and different ways of doing things, so you can talk to someone about what they do and then that might work for you and someone else might have something different to offer, so that’s what’s good about a group environment,” Parent, FG4.*

With respect to specific guidance on child behavioural issues, parents expressed some faith in government web sites and parenting sites that they trusted, such as Raising Children Network (rasingchildren.net.au). Some also mentioned “Dr Google”, Facebook parent groups, or parent blogs, but they had lower levels of trust in this information.

*“It’s hard to know what’s true and what’s not, because the internet is full of rubbish.” Parent FG3.*

The source of information most valued was advice from other mothers, especially those at playgroup. The mothers also talked about the benefits of being able to observe other parents interacting with their child at playgroup. This included observing older children, to gain an insight into what to expect when their own child reached that developmental stage.

*“I struggled a lot for a long time, but the supportive network at playgroup was good, where you looked to everyone for ideas and different approaches.” Parent, FG4.*

*Barrier Theme 1: Lack of empowerment to influence child preferences.*

A potential barrier to parental self-efficacy to implement supportive parenting practices was the parent’s perception of the child’s preferences with respect to food and activity. A number of parents made statements, including comparisons between siblings, indicating they believed their child’s preferences were fixed, and that this reduced their ability to influence their child.

*“We know that we should maybe bring a little bit more veggies or that, but we’re also limited by what they will take.” Parent, FG3.*

Some parents who felt their child would benefit from additional physical activity appeared to be constrained by their perceptions about their child’s lack of interest or enjoyment of active play.

*“I’d just like him to do it himself. Just, you know, go, ‘I’m going to go outside and play’. He’s not one of those, but he’s never been one of those kids …*” *Parent, FG3.*

Parents also generally expressed a low level of self-efficacy with respect to implementing strategies to enforce bed times and to influence the amount of child sleep. They considered child sleep issues essentially out of their control, so that even if they were aware that their child did not get enough sleep, they did not feel they could do anything about it.

*“You can recommend a certain amount of sleep, but you can’t make that happen necessarily. So sometimes, it’s like, oh that’s great I’m glad you recommend that (laughter) but good luck with that (laughter). I don’t know how that’s going to happen.” Parent, FG4.*

*Barrier Theme 2: Stress, tiredness or lack of time can make parenting a challenge.*

Throughout all of the focus group discussions, the parents made comments that demonstrated various feelings of stress and frustration in respect to day to day parenting.

*“The reality is you’re just too exhausted. You’re just surviving … I’ve always loved cooking and I’ve noticed over the last few years, I don’t enjoy it nearly as much as I used to, and it’s just simply, I still do it with this air of, ‘Oh, it’s another job to do.’ That’s unfortunate because I’m just tired and I’m just stretched.” Parent, FG5.*

A number of parents commented that they were aware that their emotions influenced their behaviour in moments of stress. They wanted to be the best parent possible, so they strived to curb behaviours they believed were detrimental to supportive parenting. Parents stated that the many demands on them as parents over the course of the day made it difficult to “cope” or deal with challenges as they arose, particularly at the end of the day.

*“I find that I need help with my emotions, I think, rather than my kids.*

*… when I get frustrated I tend to cry … By the end of the day I’m just like, oh my god. Sometimes, it just, you feel like you’ve been shouting all day.” Parent, FG3.*

Some parents expressed feelings of guilt and inadequacy.

*“You feel like the worst mother in the world.” Parent, FG1.*

*“We all kind of know the do’s and don’ts, and we all know when we do it and don’t do it and we feel the guilt for not doing them if we’re not doing them.” Parent, FG5.*

Parents also expressed not having enough time, or being too tired to use supportive parenting practices.

*“And [the parent websites] got all these mums, that got all these activities, every day and they do this and they do that. And I’m just like, I need some chill time for myself too. I mean who cleans their houses?” Parent FG3.*

### Parent preferences for a playgroup intervention

Participants were initially sceptical when asked whether a program for parents might work at their playgroup. Many stated that they either did not want to attend a program at all, or could not imagine it fitting into the noise and “chaos” of playgroup. In addition, they were not in favour of a program run outside of playgroup time (for example, in the evening) without the children in attendance. Four main themes were identified: Parents 1) come to playgroup for support and social interaction, 2) don’t want to be “educated” about parenting; they just want support, 3) feel child interruptions and distractions are unavoidable, and 4) are interested in a parent program, but don’t attend playgroup every week.

#### Theme 1: playgroup for support and social interaction

The community playgroup environment is relaxed and relatively unstructured, whereby neither adults nor children are obliged to take part in any specific activities. Parents did not want to lose that aspect of playgroup. Overwhelmingly, parents and carers attend playgroup for social interaction and to receive support from other parents, so there was some concern that any formal program would negatively impact this.

*“This is probably one of the few places where I can come, and I can just leave him, because there’s nowhere he can go, there’s little he can destroy, and I can just either sit on the steps by myself and stare at nothing, or talk to other mums.” Parent, FG5.*

However, despite these reservations, many parents were generally positive about an intervention that supported parents being delivered at playgroup.

*“I think you’re on the right track with integrating it, if that’s what you’re trying to pursue, within the framework of something that’s already happening, and that people like us are going to be at anyway. That way, if someone does want to take advantage of whatever is happening it’s not going to a thing to do it.” Parent, FG4.*

#### Theme 2: Don’t want to be “educated” about parenting; just want support

In addition to being concerned that a playgroup intervention might undermine the playgroup environment, parents were also clear that they did not want to be told what they should be doing. Rather, they wanted support and validation as a parent, as well as some useful strategies for dealing with challenging situations.

*“And probably what would be more helpful, for people that are already coming to things like playgroup, and are already seeking the best for their kids, is more of the support for the parents. It’s great to know that information, but I think a lot of it’s already, we know, like know that stuff. Like we know we shouldn’t be bribing, we know that they should be sleeping more than they are, and probably it’s more the support to help us get the best of our own situation.” Parent, FG3.*

In keeping with the theme of valuing peer support, when asked if they would prefer a health professional or a trained parent, there wasn’t a clear preference for either-- but there was unequivocal agreement that the facilitator must be a parent who understands their parenting challenges.

#### Theme 3: child interruptions and distractions are unavoidable

A fundamental feature of a playgroup is that the parent/carer and child attend together. While parents are responsible for their own child, there is an unspoken expectation that other adults will take an interest in all of the children; to supervise, intervene in child disputes, or soothe an upset child, where needed. Although children attending playgroup can, and do, mostly engage in group play with very minimal supervision, parents stated that children will often interrupt adult conversations.

*“At any moment, my child’s gonna run out and want me.” Parent 1, FG2.*

*“With the kids, it’s really impossible to sit down and have a full conversation.” Parent 2, FG2.*

A number of parents indicated that a flexible intervention, where attendees could “dip in and out” could mitigate any interruptions. When it was put to the focus groups that an option might be a formal “child minding” arrangement with child activities, almost all participants were against this idea unless it fit in with the usual environment and flow of the playgroup.

#### Theme 4: interested in a parent program, but don’t attend playgroup every week

Not all parents attend playgroup consistently, and the attendance numbers tend to vary each week. However, there was a suggestion that, if the topics were “stand alone”, then parents could specifically attend playgroup on the days when a topic was of interest. There were mixed opinions about how often a program should run, ranging from every week to every month. The underlying theme was that an intervention would need to be flexible to take into account the irregular attendance of some parents.

## Discussion

The objective of this study was to gain an understanding of the barriers and facilitators to autonomy supporting parenting practices with respect to obesity-related behaviours in children. Parents openly discussed barriers related to encouraging healthy behaviours in their children, although they generally felt they had sufficient knowledge around what those behaviours should be. They provided insights into the challenges of parenting, and the difficulties in engaging in autonomy supporting practices in the moment of feeling stressed, overwhelmed, tired or time restricted. Participants also provided insights into the barriers and facilitators to an intervention for parents in a community playgroup setting, and their preferences for mode of delivery.

Consistent with the results from other studies [37, 38], managing child food refusal through the use of non-responsive feeding practices, such as hiding vegetables, using food bribes, or only providing foods they know their child will eat, was common [39]. The use of non-responsive feeding practices has been linked to a decrease in child self-regulation and satiety responsiveness [40, 41]. In addition, the anxiety and frustration around food refusal also impacts on the maternal emotional state [42]. In the current study, most parents felt bribing children with chocolate, for example, was justified because it meant the child ate their vegetables, or finished their main meal. However, some expressed the view that, although they used this strategy, they knew it was not ideal. The use of dessert or chocolate as an incentive, and parent’s feelings of guilt about doing so, is widely reported amongst parents of young children [43, 44].

Parents discussed similar trade-offs with respect to their parenting practices around their child’s screen time. Limiting screen time is a challenge for many parents for a range of reasons, many of which relate to its appeal as a babysitter [45]. Parents talked about iPads®, iPhones® and hand-held computer games being particularly useful to occupy or distract their child due to their portability and convenience outside the home [46]. Parents felt guilty for using electronic media in this way, because they believed screen time should be restricted for children. However this attitude was undermined by the parallel belief that iPads® and computers are not only ubiquitous, but also necessary for children to master before starting school.

For most parents, physical activity was not a high priority as they felt their child was sufficiently active. Other studies have found that parents of young children often believe that children are inherently active, [45, 47]. This is a potential barrier for an intervention aiming to increase physical activity in young children [47, 48], and is supported by research that found parents feel that the physical activity guidelines apply to “other” families [49]. Conversely, some parents described their child as “not active” and stated their belief that their child’s preference for sedentary play was fixed, and they were powerless to influence this preference. Another barrier to increasing physical activity was the need to supervise the activity, either at a park or when the child was playing in the backyard at home. Parents in another qualitative study also cited safety concerns in terms of children needing to be supervised in a public location [45].

Parents felt frustrated about bed time and sleep, and believed that this was out of their control. They discussed strategies they had tried, mostly with limited success, or which impacted on themselves or their family in other ways. Consistent with other studies, parents cited daytime naps, and arriving home from work late and wanting to spend time with their children, as reasons for inconsistent bedtimes [39].

Playgroups are an important source of social support and friendship for parents, especially for those who are socially isolated [50], and they provide parents with a sense of belonging and validation as a parent [50, 51]. All parents endorsed the importance of the social support they received at playgroup. They discussed the benefits of being able to talk about their parenting challenges in an environment where the other parents understood, could offer genuine support and also suggest strategies that might help with specific issues. An intervention program that leverages this supportive environment and enables parents to share and discuss positive and responsive parenting practices therefore may be effective [39].

Parents were supportive of a program that could help them deal with the challenges of parenting, but they did not want to lose the social and informal aspects of playgroup. As such, an intervention would need to be brief, flexible and supportive. It would need to be delivered by someone they could relate to, and whom they felt would understand their parenting challenges. They commented that conversations with other parents are often interrupted by their child, or that they may be distracted by what their child is doing. However, they also indicated that they were accustomed to having disrupted conversations, so the presence of children may not be a barrier to effective implementation.

A strength of this study was the use of focus groups to explore the views of parents, allowing them to build on the views and experiences of the other parents during the discussions [52]. Another strength was the use of Social Cognitive Theory and Self-Determination Theory as conceptual frameworks. A deductive approach was taken initially in this study but then a more inductive approach was used to refine the codes and themes that emerged from the focus group discussions. This flexible analysis method enabled the research questions and aims of the study to be fully explored without being constrained by the conceptual framework.

A limitation of the study is that focus group data can only represent the views of the study participants, which may not reflect the views of a wider group of playgroup parents [53]. Even though we reached a saturation of opinions and preferences, focus groups cannot provide information about the prevalence of those opinions across the entire playgroup community [53]. Further, the playgroups that expressed an interest in taking part in the focus groups were all located in metropolitan areas of mid to high socio-economic advantage. As such, the results may not fully apply to playgroups and parents in lower socio-economic areas or to those located in regional cities or rural areas of Queensland. Another limitation of focus group data is that there may be some social desirability attached to the responses [54]. This may occur, for example, when a parent may not want their parenting challenges to be subject to judgment by other parents, or they may just conform to the general consensus of the group’s opinion [53]. This potential limitation was mitigated by the fact that the parents in each group had already established supportive and non-judgmental relationships.

## Conclusions

Parents provided insights into the challenges of parenting, and the difficulties in engaging in autonomy supporting parenting practices when feeling stressed, overwhelmed, tired or time restricted. Childhood obesity prevention interventions targeting parenting practices related to healthy lifestyle behaviours thus need to be implemented in a way that supports parents, increases parental self-efficacy, and decreases parental stress. The community playgroup environment is mostly unstructured, often noisy, and conversations are frequently interrupted by the needs of the children. As such, any obesity prevention program implemented in this setting would need to be light touch, flexible, and where possible, facilitated by a peer. Studies exploring the feasibility and potential efficacy of a peer-facilitated childhood obesity prevention intervention, delivered in a community playgroup setting, are thus warranted.

## Supplementary information

**Additional file 1.** Focus group topic guide.

## Data Availability

The transcripts analysed during this study are available from the corresponding author on reasonable request.

## Abbreviations

FG: Focus Group
PGQ: Playgroup Queensland

## References

[1] Playgroup Australia Limited (2015). Playgroup Australia Limited: Annual Report.

[2] World Health Organisation (2017). Report of the Commission on Ending Childhood Obesity. Implementation plan: Executive summary. Geneva: WHO.

[3] Leech RM, McNaughton SA, Timperio A (2014). The clustering of diet, physical activity and sedentary behavior in children and adolescents: a review. Int J Behav Nutr Phys Act.

[4] Skouteris H, McCabe M, Swinburn B, Newgreen V, Sacher P, Chadwick P (2011). Parental influence and obesity prevention in pre-schoolers: a systematic review of interventions. Obes Rev.

[5] Lindsay AC, Sussner KM, Kim J, Gortmaker S (2006). The role of parents in preventing childhood obesity. Futur Child.

[6] Power TG, EFC S, Berge J, Connell L, Govig B, Hennessy E (2013). Contemporary research on parenting: Conceptual, methodological, and translational issues. Child Obes.

[7] Patrick H, Hennessy E, McSpadden K, Oh A (2013). Parenting styles and practices in children's obesogenic behaviors: Scientific gaps and future research directions. Child Obes.

[8] Yee AZH, Lwin MO, Ho SS (2017). The influence of parental practices on child promotive and preventive food consumption behaviors: a systematic review and meta-analysis. Int J Behav Nutr Phys Act.

[9] Xu Huilan, Wen Li Ming, Rissel Chris (2015). Associations of Parental Influences with Physical Activity and Screen Time among Young Children: A Systematic Review. Journal of Obesity.

[10] Côté-Lecaldare M, Joussemet M, Dufour S (2016). How to support toddlers' autonomy: a qualitative study with child care educators. Early Educ Dev.

[11] Hurley KM, Cross MB, Hughes SO (2011). A systematic review of responsive feeding and child obesity in high-income countries. J Nutr.

[12] Trost SG, McDonald S, Cohen A (2013). Measurement of general and specific approaches to Physical Activity Parenting: A systematic review. Child Obes.

[13] TM O’C, Hingle M, Chuang R-J, Gorely T, Hinkley T, Jago R (2013). Conceptual understanding of screen media parenting: Report of a working group. Child Obes.

[14] Mindell JA, Williamson AA (2018). Benefits of a bedtime routine in young children: sleep, development, and beyond. Sleep Med Rev.

[15] Paes VM, Ong KK, Lakshman R (2015). Factors influencing obesogenic dietary intake in young children (0-6 years): systematic review of qualitative evidence. BMJ Open.

[16] Grossklaus H, Marvicsin D (2014). Parenting efficacy and its relationship to the prevention of childhood obesity. Pediatr Nurs.

[17] Gerards SM, Sleddens EF, Dagnelie PC, de Vries NK, Kremers SPJ (2011). Interventions addressing general parenting to prevent or treat childhood obesity. Int J Pediatr Obes.

[18] Golley RK, Perry RA, Magarey A, Daniels LA (2007). Family-focused weight management program for five- to nine-year-olds incorporating parenting skills training with healthy lifestyle information to support behaviour modification. Nutr Diet.

[19] Magarey A, Perry RA, Baur LA, Steinbeck KS, Sawyer M, Hills AP (2011). A parent-led family-focused treatment program for overweight children aged 5 to 9 years: the PEACH RCT. Pediatrics..

[20] Ash T, Agaronov A, Young T, Aftosmes-Tobio A, Davison KK (2017). Family-based childhood obesity prevention interventions: a systematic review and quantitative content analysis. Int J Behav Nutr Phys Act.

[21] Hnatiuk J. A., Brown H. E., Downing K. L., Hinkley T., Salmon J., Hesketh K. D. (2018). Interventions to increase physical activity in children 0–5 years old: a systematic review, meta‐analysis and realist synthesis. Obesity Reviews.

[22] Taylor B, Gray AR, Galland BC, Heath A-LM, Lawrence J, Sayers RM (2017). Targeting sleep, food, and activity in infants for obesity prevention: an RCT. Pediatrics..

[23] Waters E, de Silva-Sanigorski A, Hall BJ, Brown T, Campbell KJ, Gao Y, et al. Interventions for preventing obesity in children. Cochrane Database Syst Rev. 2011;7(12):CD001871. 10.1002/14651858.CD001871.pub3. 10.1002/14651858.CD001871.pub3 22161367

[24] Matvienko-Sikar K, Toomey E, Delaney L, Harrington J, Byrne M, Kearney PM (2018). Effects of healthcare professional delivered early feeding interventions on feeding practices and dietary intake: a systematic review. Appetite..

[25] Bell L, Golley RK (2015). Interventions for improving young children’s dietary intake through early childhood settings: a systematic review. Int J Child Health Nutr.

[26] Morris H, Skouteris H, Edwards S, Rutherford L (2015). Obesity prevention interventions in early childhood education and care settings with parental involvement: a systematic review. Early Child Dev Care.

[27] Strange C, Fisher C, Howat P, Wood L (2014). Fostering supportive community connections through mothers' groups and playgroups. J Adv Nurs.

[28] Australia P (2017). Playgroup Australia: annual report.

[29] Queensland P (2018). Playgroup Queensland: annual report.

[30] McLean K, Edwards S, Evangelou M, Skouteris H, Harrison LJ, Hemphill SA (2017). Playgroups as sites for parental education. J Early Child Res.

[31] Parker A, Tritter J (2006). Focus group method and methodology: current practice and recent debate. Int J Res Method Educ.

[32] Elo S, Kyngäs H (2008). The qualitative content analysis process. J Adv Nurs.

[33] Ryan RM, Deci EL (2000). Self-determination theory and the facilitation of intrinsic motivation, social development, and well-being. Am Psychol.

[34] Ryan RM, Deci EL (2017). Self-determination theory: basic psychological needs in motivation, development, and wellness.

[35] Bandura A (1986). Social foundations of thought and action: a social cognitive theory.

[36] Bengtsson M (2016). How to plan and perform a qualitative study using content analysis. NursingPlus Open.

[37] Petrunoff NA, Lwilkenfeld R, King LA, Flood VM (2014). ‘Treats’, ‘sometimes foods’, ‘junk’: a qualitative study exploring ‘extra foods’ with parents of young children. Public Health Nutr.

[38] Jarman M, Ogden J, Inskip H, Lawrence W, Baird J, Cooper C (2015). How do mothers manage their preschool children's eating habits and does this change as children grow older? A longitudinal analysis. Appetite..

[39] Martin-Biggers J, Spaccarotella K, Hongu N, Alleman G, Worobey J, Byrd-Bredbenner C (2015). Translating it into real life: a qualitative study of the cognitions, barriers and supports for key obesogenic behaviors of parents of preschoolers. BMC Public Health.

[40] Francis LA, Susman EJ (2009). Self-regulation and rapid weight gain in children from age 3 to 12 years. Arch Pediatr Adolesc Med.

[41] Eneli IU, Crum PA, Tylka TL (2008). The trust model: a different feeding paradigm for managing childhood obesity. Obesity..

[42] Mitchell G, Farrow C, Haycraft E, Meyer C (2013). Parental influences on children's eating behaviour and characteristics of successful parent-focussed interventions. Appetite..

[43] Campbell K, Crawford DA, Hesketh KD (2007). Australian parents’ views on their 5-6-year-old children’s food choices. Health Promot Int.

[44] Tucker P, Irwin JD, He M, Sangster Bouck LM, Pollett G (2006). Preschoolers’ dietary behaviours: Parents’ perspectives. Can J Diet Pract Res.

[45] Hesketh KD, Hinkley T, Campbell KJ (2012). Children′s physical activity and screen time: qualitative comparison of views of parents of infants and preschool children. Int J Behav Nutr Phys Act.

[46] Bentley GF, Turner KM, Jago R. Mothers views of their preschool child's screen-viewing behaviour: A qualitative study. BMC Public Health. 2016;16:718. 10.1186/s12889-016-3440-z. 10.1186/s12889-016-3440-z PMC4973523 27492488

[47] Hennink-Kaminski H, Ihekweazu C, Vaughn AE, Ward DS. Using formative research to develop the healthy me, healthy we campaign: partnering childcare and home to promote healthy eating and physical activity behaviors in preschool children. Soc Mark Q. 2018;24(3):194–215.

[48] Hesketh KR, McMinn A, Griffin S, Harvey N, Godfrey K, Inskip H (2013). Maternal awareness of young children's physical activity: levels and cross-sectional correlates of overestimation. BMC Public Health.

[49] Bentley Georgina F, Jago Russell, Turner Katrina M (2015). Mothers’ perceptions of the UK physical activity and sedentary behaviour guidelines for the early years (Start Active, Stay Active): a qualitative study. BMJ Open.

[50] Hancock KJ, Cunningham NK, Lawrence D, Zarb D, Zubrick SR (2015). Playgroup participation and social support outcomes for mothers of young children: a longitudinal cohort study. PLoS One.

[51] Harman B, Guilfoyle A, O’Connor M (2014). Why mothers attend playgroup. Australas J Early Childhood.

[52] De Decker E, De Craemer M, De Bourdeaudhuij I, Wijndaele K, Duvinage K, Koletzko B (2012). Influencing factors of screen time in preschool children: an exploration of parents' perceptions through focus groups in six European countries. Obes Rev.

[53] Liamputtong P (2013). Qualitative research methods.

[54] Bellows L, Anderson J, Gould SM, Auld G (2008). Formative research and strategic development of a physical activity component to a social marketing campaign for obesity prevention in preschoolers. J Community Health.

