# Supplementary material for: Supporting healthy lifestyle behaviours in families attending community playgroups: parents’ perceptions of facilitators and barriers
Source: BMC Public Health. 2019 Dec 27;19:1740. doi: 10.1186/s12889-019-8041-1 (PMC6935103; doi:10.1186/s12889-019-8041-1)
Supplement: Supplementary file 1 — Additional file 1. Focus group topic guide. [file 12889_2019_8041_MOESM1_ESM.docx]

**Focus Groups with parents at playgroup – Topic Guide**

Thanks for coming along today. We’re here to talk about what kind of information and support parents want about their child’s health. I’m going to ask questions about food, active play, screen time and sleep. You don’t have to answer a question if you don’t want to, but I am hoping we can bounce some ideas around as a group and you can tell me what you think.

| **Focus group question** | **Prompts** |
| --- | --- |
| 1. **Can you tell me what you enjoy about coming to playgroup?** | - How is the playgroup run? - What do you like about playgroup? - What do you get out of playgroup? |
| 1. **Where do you get information about healthy child behaviours?** | - Food, eating - Active play / energetic play - Screen time (TV, iPads, computers, hand-held games) - Child development / parenting - How do you know if the information is reliable? - Have you been to any programs run by playgroups? - Have you been to any programs for parents outside playgroup? |
| 1. **What barriers do you face when it comes to encouraging healthy behaviours in your child?** | - What food to provide / How much to provide? - Are there any foods or drinks that concern you or that you try to limit? Do you find it hard to limit these foods? - What influences the type of food and drink? - What is it about your child that influences what you feed them? - Being active enough - Limiting screen time - Getting sufficient sleep and a regular bedtime - Barriers that impact across all 4 behaviours |
| 1. **What things are helpful when it comes to encouraging healthy behaviours in your child?** | - What tips and strategies work when it comes to influencing:   - What and how much your child eats?   - What they play?   - How much they watch TV or use electronic devices?   - When they go to bed?   - Things that influence all 4 behaviours? - What information do you want or need about your child’s: food and eating behaviours? PA? Screen time? Sleep? - Do you find discussing concerns around your child’s health or behaviours with other parents helpful? - What type of parenting information or strategies would be helpful to you? |
| 1. **How do you think a healthy lifestyle program at playgroup could work?** | - How could it be run in playgroup time? - What issues might there be in running a program for parents at playgroup? - What would work well? - How could it be incorporated into the playgroup schedule? - Would it be better to run it separately from your usual playgroup time slot? - Who should deliver the program? A parent? Health professional? - Would you prefer weekly or fortnightly or some other timeframe? - What format would be ideal for supporting information (hard copy, website or app)? |
